# A case of hepatic pseudolymphoma in a patient with primary biliary cirrhosis

**DOI:** 10.1002/ccr3.2378

**Published:** 2019-08-20

**Authors:** Masashi Inoue, Masahiro Tanemura, Tomio Yuba, Tatsuya Miyamoto, Megumi Yamaguchi, Toshimitsu Irei, Shingo Seo, Toshihiro Misumi, Wataru Shimizu, Takahisa Suzuki, Takashi Onoe, Takeshi Sudo, Yosuke Shimizu, Takao Hinoi, Hirotaka Tashiro

**Affiliations:** ^1^ Department of Surgery National Hospital Organization Kure Medical Center Chugoku cancer center Kure Japan; ^2^ Department of Surgery Osaka Police Hospital Osaka Japan; ^3^ Present address: Department of Surgery National Hospital Organization Higashihiroshima Medical Center Higashihiroshima Japan

**Keywords:** biliary cirrhosis, hepatic pseudolymphoma, hepatocellular carcinoma

## Abstract

Hepatic pseudolymphoma is a very rare benign reactive lymphoid hyperplasia associated with autoimmunity and chronic inflammatory liver diseases such as primary biliary cirrhosis and may mimic hepatocellular carcinoma. This diagnosis should be suspected in female with a suspicious single tumor. Close monitoring is needed in view of its premalignant nature.

## 1 BACKGROUND

Pseudolymphoma is a rare disease that forms a mass‐like lesion and is characterized by the proliferation of non‐neoplastic, polyclonal lymphocytes forming follicles with an active germinal center.^1^ Pseudolymphoma is also termed reactive lymphoid hyperplasia or nodular lymphoid lesion. Pseudolymphoma is most commonly described in the skin 2 and gastrointestinal tract.^3^, ^4^ Hepatic pseudolymphoma (HPL) is an extremely rare disease, and it is very difficult to distinguish HPL from other malignant diseases, such as hepatocellular carcinoma (HCC), based on imaging examinations. HPL is often confirmed and diagnosed based on postoperative pathologic evaluation. Even though the etiology and pathogenesis of HPL are unknown, the association with a chronic infection or inflammatory process suggests an association with an immunologic response.^5^ We describe a case of HPL associated with primary biliary cirrhosis (PBC) and review the literature to reveal the clinicopathologic characteristics.

## 2 CASE PRESENTATION

A 70‐year‐old woman was shown to have a mass, 10 mm in diameter, in segment Ⅷ of the liver during a follow‐up evaluation for PBC. Her social and family histories were unremarkable. She had chronic rheumatoid arthritis in addition to PBC, and she was prescribed oral steroids and methotrexate. She had no abnormal physical findings.

Laboratory testing was negative for hepatic virus, and hepatic function was in the normal range, although the antimitochondrial antibody titer was positive. Tumor markers, including carcinoembryonic antigen, carbohydrate antigen 19‐9, alpha‐fetoprotein (AFP), and protein induced by vitamin K absence or antagonist Ⅱ(des‐gamma‐carboxy prothrombin [PIVKA‐Ⅱ]), were within normal limits. In addition, the ICG 15 min value was 9.8% and the Child‐Pugh classification was A at 5 points (Table 1).

**Table 1:** Laboratory data

〈complete blood count〉								
WBC	6300	/uL	UA	6.2	mg/dL	HBsAg	−	
HGB	14.3	g/dL	T‐Bil	0.7	mg/dL	HBsAb	＋	
Neut%	53.2	%	D‐Bil	0.1	mg/dL	HCVAb	−	
PLT	17.9	×10^4^/uL	TP	7.2	g/dL	Antinuclear antibody	<2.0	
〈biological examination〉			Alb	4.3	g/dL	Antimitochondrial antibody	68	U/mL
Na	139	mEq/L	T‐cho	204	mg/dL	AFP	14	ng/mL
Cl	104	mEq/L	TG	131	mg/dL	PIVKA‐II	29	mAU/mL
K	4.3	mEq/L	HDL‐C	63	mg/dL	CEA	<0.5	ng/mL
AST	35	IU/L	LDH‐C	113	mg/dL	CA19‐9	3	U/mL
ALT	45	IU/L	CRP	0.34	mg/dL	〈Blood coagulation test〉		
LDH	172	IU/L	IgG	1190	mg/dL	PT%	103.9	%
ALP	233	IU/L	IgA	224	mg/dL	APTT	26	Sec
γ‐GTP	143	IU/L	IgM	47	mg/dL	Fib	245.3	mg/dL
Ch‐E	347	IU/L	IgE	6.9	K/U	FDP	0.1	μg/dL
BUN	15	mg/dL						
CRE	0.34	mg/dL				ICG 15 min	9.8	%

Note

Laboratory testing was negative for hepatic virus and hepatic function was in the normal range, although the antimitochondrial antibody titer was positive. Tumor markers, including carcinoembryonic antigen, carbohydrate antigen 19‐9, alpha‐fetoprotein (AFP), and protein induced by vitamin K absence or antagonist Ⅱ(des‐gamma‐carboxy prothrombin [PIVKA‐Ⅱ]), were within normal limits. In addition, the ICG 15 min value was 9.8% and the Child‐Pugh classification was A at 5 points.

Abbreviations: AFP, α‐fetoprotein; Alb, albumin; ALP, alkaline phosphatase; ALT, alanine aminotransferase; APTT, activated partial thromboplastin time; AST, aspartate aminotransferase; BUN, blood urea nitrogen; CA19‐9, carbohydrate antigen 19‐9; CEA, carcinoembryonic antigen; Ch‐E, cholinesterase; Cl, chlorine; Cr, creatinine; CRP, c‐reactive protein; D‐bil, direct bilirubin; FDP, fibrin and fibrinogen degradation products; Fib, fibrinogen; HBsAb, hepatitis B surface antibody; HBsAg, hepatitis B surface antigen; HCVAb, hepatitis C virus antibody; HDL‐C, high‐density lipoprotein cholesterol; HGB, hemoglobin; ICG, indocyanine green; IgA, immunoglobulin A; IgE, immunoglobulin E; IgG, immunoglobulin G; IgM, immunoglobulin M; K, potassium; LDH, lactate dehydrogenase; LDH‐C, low‐density lipoprotein cholesterol; Na, sodium; Neut, neutrophil; PIVKA‐Ⅱ, protein induced by vitamin K absence or antagonist‐Ⅱ; Plt, platelet; PT, prothrombin time; T‐bil, total bilirubin; T‐cho, total cholesterol; TG, triglyceride; TP, total protein; UA, uric acid; WBC, white blood cell; γ‐GTP, γ‐glutamyltransferase.

**Figure 1:**
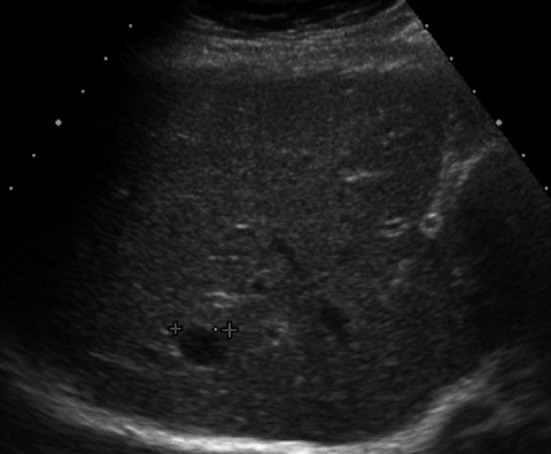
Abdominal ultrasonography showed a hypoechoic lesion, 13.4 mm in diameter, in segment 8 in the liver

**Figure 2:**
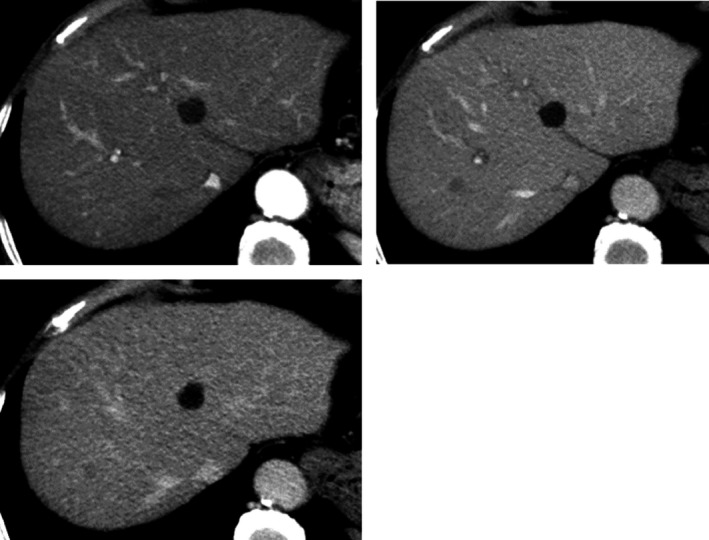
On abdominal computed tomography (CT) scan, a mass, 10 mm in diameter, was demonstrated, which was slightly enhanced in the early arterial phase and subsequently washed out in the late phase after contrast material injection, but was not consistent with a HCC. Other organs, including regional or para‐aortic lymph nodes, showed no abnormal findings

**Figure 3:**
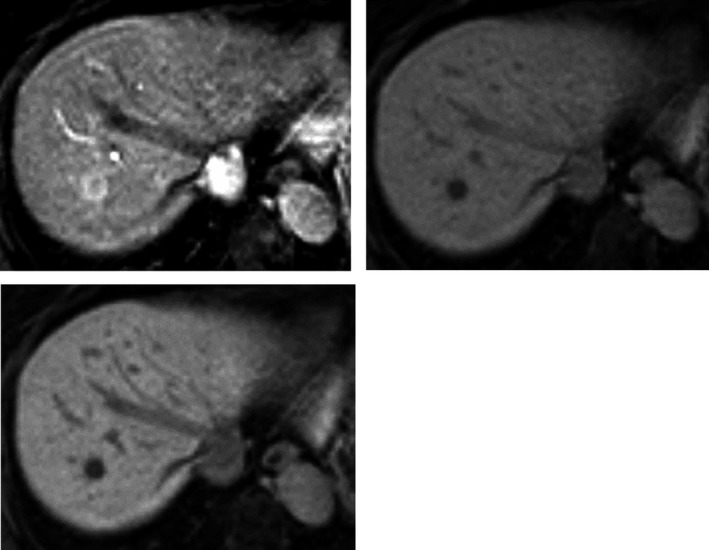
On gadoxetic acid (Gd‐EOB‐DTPA)‐enhanced magnetic resonance imaging (MRI), abbreviated as EOB‐MRI, the mass was enhanced in the arterial dominant phase and washed out in the late phase and hepatocyte phase

Abdominal ultrasonography showed a hypoechoic mass, 13.4 mm in diameter, in segment Ⅷ of the liver (Figure 1). An abdominal computed tomography (CT) scan showed a mass, 10 mm in diameter, which was slightly enhanced in the early phase and washed out in the late phase (Figure 2). On gadoxetic acid (Gd‐EOB‐DTPA)‐enhanced magnetic resonance imaging (MRI), the mass was enhanced in the arterial dominant phase and washed out in the late and hepatocyte phases (Figure 3).

Based on the preoperative diagnosis of HCC, a laparoscopic‐assisted S8 subsegmentectomy was planned. The operation began with laparoscopic right liver mobilization with the camera port and three ports under the right brow arch, followed by right subcostal oblique incision for hepatic resection.

**Figure 4:**
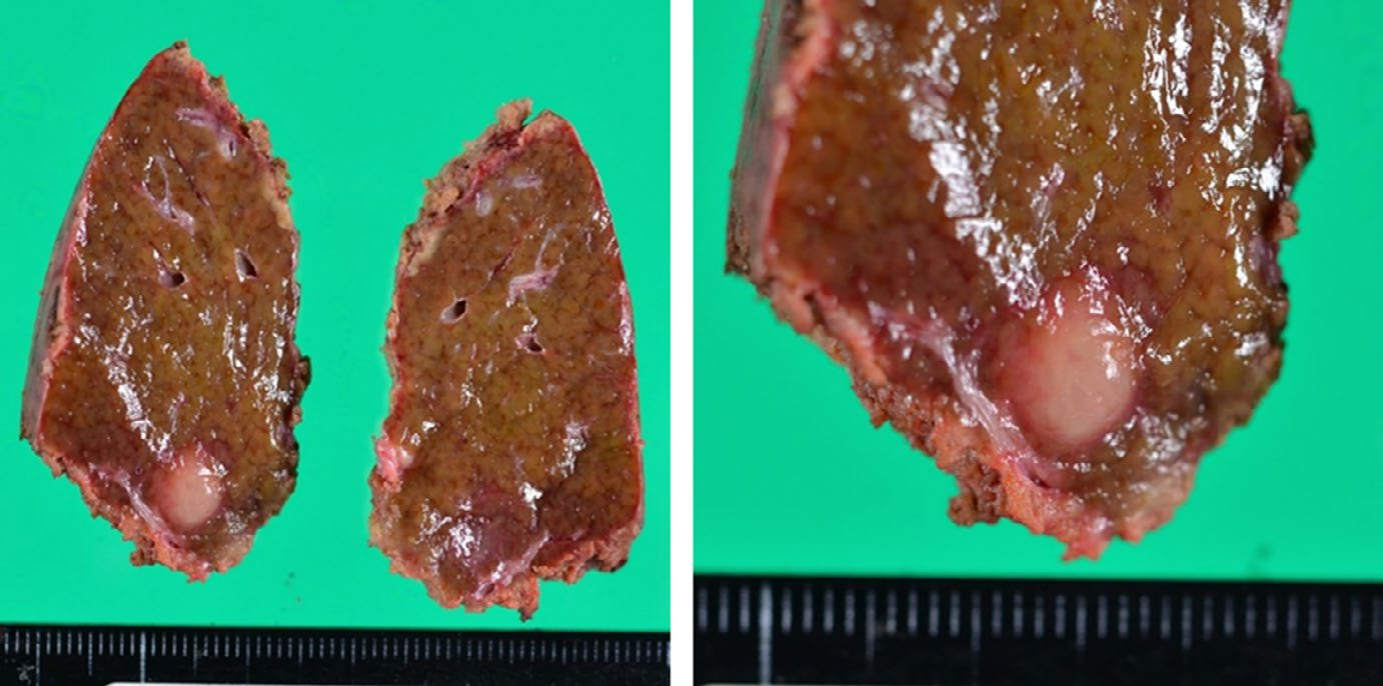
On macroscopic examination, there was a gray‐white solid tumor measuring 9 mm in the largest diameter. The tumor was completely excised

**Figure 5:**
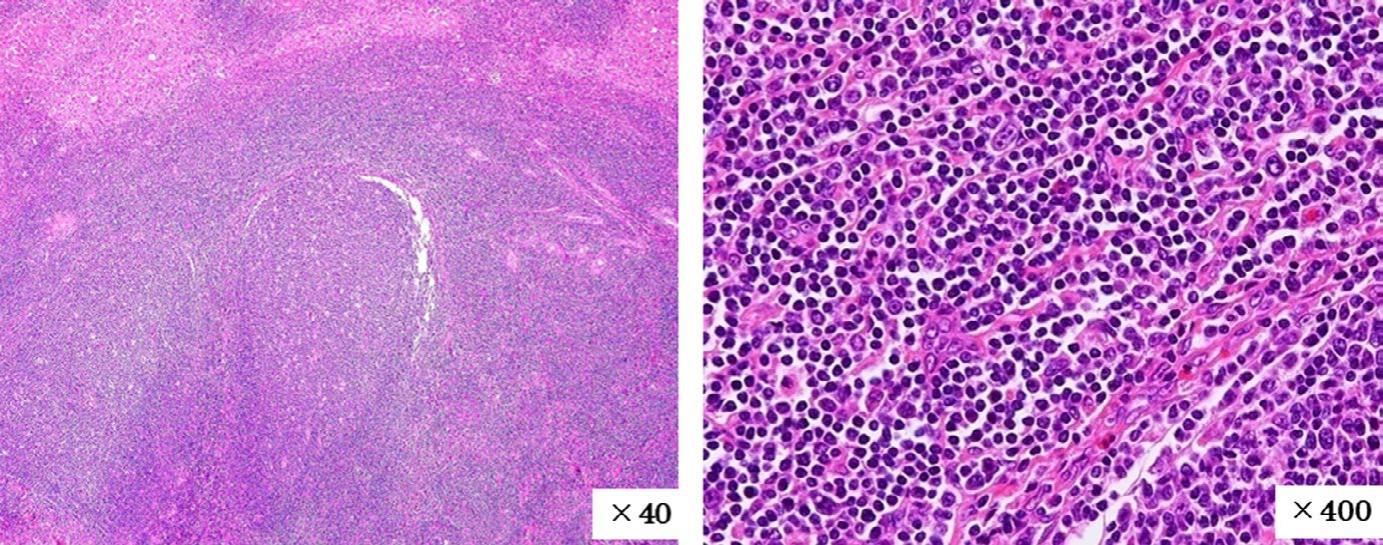
On microscopic examination, the tumor in the liver was composed of dense lymphocytic infiltration, including multiple lymphoid follicles with germinal centers. The interfollicular areas were expanded and filled with small‐to‐medium lymphocytes without cellular atypia

A gray‐white solid tumor with a maximum diameter of 9 mm was observed macroscopically (Figure 4). The tumor in the liver consisted of a dense lymphocytic infiltration, including multiple lymphoid follicles with germinal centers, microscopically. The interfollicular areas were expanded and filled with small‐to‐medium lymphocytes without cellular atypia (Figure 5).

**Figure 6:**
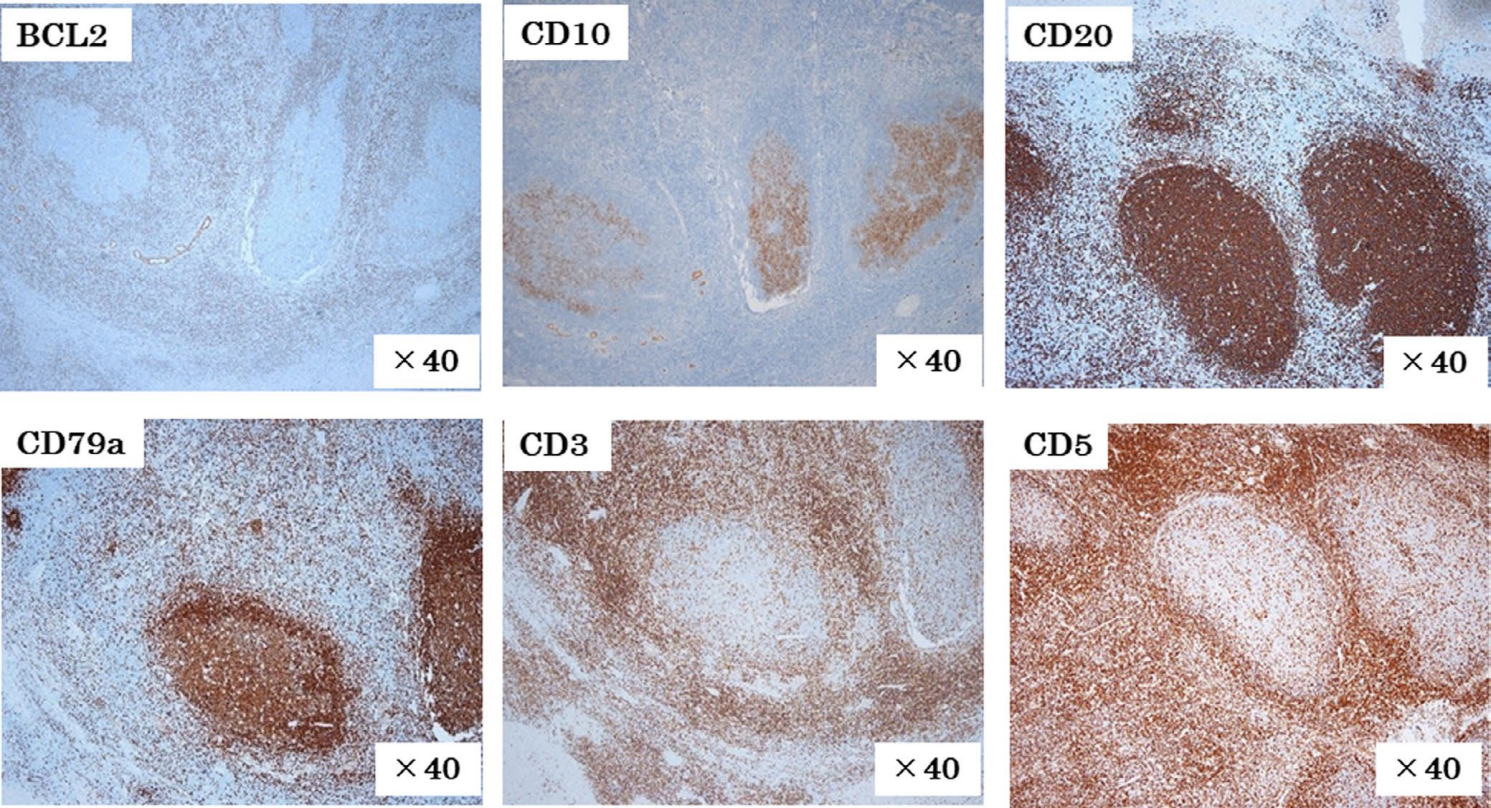
Immunohistochemical staining revealed that the follicles were CD20(+), CD79a(+), CD10(+), and Bcl‐2(‐), and the interfollicular area was CD3(+) and CD5(+). Taken together, a diagnosis of pseudolymphoma was favored

Immunohistochemical staining revealed that the follicles were CD20 (+), CD79a (+), CD10 (+), and Bcl‐2 (−), and the interfollicular area was CD3 (+) and CD5 (+). Taken together, a diagnosis of pseudolymphoma was favored (Figure 6).

The postoperative course was good, and she was discharged on postoperative day 7.

## 3 DISCUSSION

Pseudolymphoma, which is also termed reactive lymphoid hyperplasia or nodular lymphoid lesion, is a rare disease, especially in the liver. Based on a review of the PubMed database from 1981 to 2017 using the keywords “pseudolymphoma,” “lymphoid hyperplasia of the liver,” and “nodular lymphoid lesion,” we found 80 cases of pseudolymphomas, including our case (Table 2). In the previously reported cases, images of HPL depicted a hypoechoic lesion on ultrasonography and varied from hyper‐ to hypovascular on contrast CT, MRI, and angiography.^6^, ^7^ The preoperative diagnosis of HPL has features similar to those of hepatocellular carcinoma. Many cases of HPL have been misdiagnosed as HCC or metastatic tumors. The preoperative diagnosis was described in 65 cases, 33 of which were diagnosed as hepatocellular carcinoma and 16 were diagnosed as metastatic liver tumors. Although the preoperative diagnosis is difficult, when examining the characteristics of HPL, 86.3% of the reports described a single tumor. The average age of the patients was 56.6 years (range = 15‐81 years). The frequency of females was greater (F:M = 74:6 [92.5%]). The average size of the tumor was 17.4 mm (range, 3‐60 mm), and 88.8% of the tumors were ≤20 mm in size.

**Table 2:** Clinical presentation of reported cases of hepatic pseudolymphomas (total n = 80)

Age		56.6(range 15‐81)	
Gender	Male	6	7.50%
	Female	74	92.50%
Associated autoimmune disease			
	Sjogren`s syndrome	3	
	Autoimmune thyroiditis	3	
	Takayasu disease	1	
	CREST syndrome	1	
Associated liver disease			
	PBC	13	
	Chronic viral hepatitis B	7	
	Chronic viral hepatitis C	2	
Preoperative diagnosis		65 described	
	HCC	33	
	Metastatic tumor	16	
	CCC	2	
	Pseudolymphoma	3	
	MALT lymphoma	2	
	others	9	
Tumor number			
	Solitary	69	
	Multiple	11	
Tumor size, mm		17.4(range 3‐60)	
Treatment		72 described	
	Resection	60	
	Transplantation	4	
	Biopsy	4	
	Others	4	

Note

We found 80 cases of pseudolymphomas, including our case. The preoperative diagnosis was described in 65 cases, 33 of which were diagnosed as hepatocellular carcinoma, and 16 were diagnosed as metastatic liver tumors. Although the preoperative diagnosis is difficult, when examining the characteristics of HPL, 86.3% of the reports described a single tumor. The average age of the patients was 56.6 years (range = 15‐81 years). The frequency of females was greater (F:M = 74:6 [92.5%]). The average size of the tumor was 17.4 mm (range, 3‐60 mm), and 88.8% of the tumors were <20 mm in size.

Abbreviations: CCC, cholangiocellular carcinoma; CREST, calcinosis, Raynaud's phenomenon, esophageal dysmotility, sclerodactyly, and telangiectasia; HCC, hepatocellular carcinoma; MALT, mucosa‐associated lymphoid tissue; PBC, primary biliary cirrhosis.

In this case, HPL appeared during the course of PBC, but there were also 13 cases (16.3%) of HPL associated with PBC (Table 3).^8^, ^9^, ^10^, ^11^, ^12^, ^13^, ^14^, ^15^, ^16^ Because the lesion had been misdiagnosed as HCC or another malignant tumor, 10 HPL patients associated with PBC underwent hepatic resections. Three tumors were found in the liver and resected by transplantation for PBC.

**Table 3:** Reported cases of hepatic pseudolymphomas in patients with PBC

Author(s)	Age	Sex	Tumor number	Tumor size(mm)	preoperative diagnosis	Treatment	Associated disease	Reference
Toshihide Okada	63	F	2	13,0,4	HCC	Resection	PBC, Primary aldosteronism	8
Yoh Zen	63	F	2	9,5		Resection	PBC, Chronic thyroiditis	9
Shin‐ichiro Sato	55	F	1	11		Needle biopsy	PBC	10
Mitsuaki Ishida	68	F	1	20	Metastasis of gastric cancer	Resection	PBC, Gastric cancer	11
Jessica Calvo	70	F	1	23		Resection	PBC	12
Jessica Calvo	80	F	1	13		Biopsy	PBC, Sjogren syndrome	12
Sheida Sharifi	52	F	1	4	PBC	Transplantation	PBC	13
Sheida Sharifi	56	F	1	15	PBC	Transplantation	PBC, CREST	13
Yuka Fukuo	47	F	2	15,5	HCC	Resection	PBC	14
Dominguez‐Perez	58	F	1	10	Malignancy, premalignancy	Resection	PBC	15
Higashi	52	F	1	16	HCC	Transplantation	PBC	16
Higashi	51	F	1	18	HCC	Resection	PBC	16
Our case	70	F	1	10	HCC	Resection	PBC, chronic rheumatoid arthritis	

Note

There were 13 cases (16.3%) of HPL associated with PBC. Because the lesion had been misdiagnosed as HCC or another malignant tumor, 10 HPL patients associated with PBC underwent hepatic resections. Three tumors were found in the liver and resected by transplantation for PBC.

Abbreviations: CREST, calcinosis, Raynaud's phenomenon, esophageal dysmotility, sclerodactyly, and telangiectasia; HCC, hepatocellular carcinoma; PBC, primary biliary cirrhosis.

Primary biliary cirrhosis is a chronic progressive cholestatic liver disease. The pathogenesis of PBC is presumed to have an underlying autoimmune mechanism. Histologically, the interlobular bile ducts are primarily damaged and show characteristic findings, such as chronic nonsuppurative destructive cholangitis (CNSDC), followed by progressive bile duct loss.^15^, ^16^ Some of the HPL patients present with extrahepatic autoimmune diseases, such as Sjogren's syndrome,^10^, ^12^, ^17^ autoimmune thyroiditis,^6^, ^9^, ^18^ Takayasu aortitis with antiphospholipid syndrome,^9^ or calcinosis, Raynaud's phenomenon, esophageal dysmotility, sclerodactyly, and telangiectasia (CREST syndrome; 13). Because HPL has a relatively large number of autoimmune disease complications, it is suggested that an autoimmune mechanism is involved in the increase in HPL.^17^, ^18^ In addition, many of the HPL patients were shown to have chronic liver diseases, such as PBC and viral hepatitis, representing 27.5% of affected patients. It is also suggested that the pathogenesis of HPL might involve a chronic reaction.^19^

In contrast, PBC is rarely complicated by HCC. According to the data of all reviewed PBC patients in Japan, the incidence of HCC was 2.4%.^20^ PBC is pathologically characterized by CNSDC, thus inflammatory lesions of PBC are mainly present on cholangiocytes, and it is thought that HCC is less frequent in PBC patients due to poor inflammation in hepatocytes, as is seen in viral infections. So, the possibility of HPL should be considered if a woman with PBC is shown to have a liver tumor <20 mm in size.

Because the diagnosis often is established from surgery, the natural history of HPL is not well understood. Malignant conversion of pseudolymphoma has been reported in the lungs, stomach, and skin.^21^, ^22^, ^23^ With respect to the liver, one case report is available,^10^ with the possibility of transformation of HPL into hepatic MALT lymphoma. This case was HPL in a patient with PBC. HPL is generally regarded as a benign disease, but because there is the possibility of malignant conversion, it is important to follow the patient closely.

## 4 CONCLUSION

Hepatic pseudolymphoma should be considered when a single small tumor is found in middle‐aged females with PBC. Considering that the real nature of HPL remains unclear to date, it is necessary to follow‐up carefully.

## CONFLICT OF INTEREST

None declared.

## AUTHOR CONTRIBUTION

MI wrote the manuscript. MI and MT designed the study. TY, TM, MY, TI, SS, TM, WS, TS, TO, TS, YS, TH, and HT, proofread the manuscript.
